# Age differences in the neural response to emotional distraction during working memory encoding

**DOI:** 10.3758/s13415-018-0610-8

**Published:** 2018-06-11

**Authors:** Maryam Ziaei, George Samrani, Jonas Persson

**Affiliations:** 1 School of Psychology, The University of Queensland, Brisbane, Australia; 2 Centre for Advanced Imaging, The University of Queensland, Brisbane, Australia; 3 Aging Research Center (ARC), Karolinska Institutet and Stockholm University, Tomtebodavägen 18A, 17165 Solna, Sweden

**Keywords:** Emotional distraction, Working memory, Interference resolution, Aging, fMRI

## Abstract

Age-related declines in attention and working memory (WM) are well documented and may be worsened by the occurrence of distracting information. Emotionally valenced stimuli may have particularly strong distracting effects on cognition. We investigated age-related differences in emotional distraction using task-fMRI. WM performance in older adults was lower for emotional compared with neutral distractors, suggesting a disproportional impairment elicited by emotional task-irrelevant information. Critically, older adults were particularly distracted by task-irrelevant positive information, whereas the opposite pattern was found for younger adults. Age groups differed markedly in the brain response to emotional distractors; younger adults activated posterior cortical regions and the striatum, and older adults activated frontal regions. Also, an age by valence interaction was found for IFG and ACC, suggesting differential modulation of attention to task-relevant emotional information. These results provide new insights into age-related changes in emotional processing and the ability to resolve interference from emotional distraction.

**Electronic supplementary material:**

The online version of this article (10.3758/s13415-018-0610-8) contains supplementary material, which is available to authorized users.

## Introduction

In daily life, we often are required to filter out irrelevant information to focus on a main task. Emotional stimuli capture attention more easily relative to neutral ones (Vuilleumier, 2005) and therefore may compete with attentional and cognitive resources involved in processing goal-relevant information. According to the dual competition model (Pessoa, 2008, 2009), emotional information receives prioritized processing, which is beneficial when the information is relevant for current goals but may be disrupting when it interferes with such goals. Biased allocation of attention to emotionally valenced, but task-irrelevant, information during concomitant task performance has been shown to impair the ability to perform a task at an optimal level (Algom, Chajut, & Lev, 2004; Hodsoll, Viding, & Lavie, 2011; Sussman, Heller, Miller, & Mohanty, 2013).

Mounting evidence suggests a role of emotional distractors and their valence on performance during different stages of the working memory (WM) tasks (Diaz et al., 2011; Dolcos & McCarthy, 2006; García-Pacios, Del Río, Villalobos, Ruiz-Vargas, & Maestú, 2015; Iordan & Dolcos, 2017; Iordan, Dolcos, & Dolcos, 2013; Oei et al., 2012). While some studies have shown a detrimental role of negatively-valenced distractors, others have shown that emotional distractors with pleasant content have less negative impact on the task, relative to neutral (García-Pacios et al., 2015) or negative stimuli (García-Pacios et al., 2015; García-Pacios, Garcés, Del Río, & Maestú, 2017). Brain imaging evidence has also shown that resolving interference from distractors may engage partially segregated neural representations—depending on the emotional valence of the distractors (Chechko et al., 2012; Compton et al., 2003; Whalen et al., 1998). For example, Anticevic et al. (2010) reported a negative relationship between the amygdala and prefrontal cortex (PFC) when negative distractors were present compared to neutral ones (Anticevic, Repovs, & Barch, 2010). Converging evidence suggests that distractors with negatively-valenced contents adversely impact performance by disrupting activity of regions associated with the “dorsal executive” neural system—a network involved in executive control—along with increased activation of regions associated with the “ventral affective” neural system—a network involved in processing emotional information.

Given that emotional distractors capture attention more easily compared to neutral distractors (Anderson, 2005; Whalen et al., 1998; Vuilleumier, 2005), suppression of task-irrelevant emotional information requires cognitive inhibitory control. Aging, however, is associated with decline of inhibitory control and interference resolution, which are essential for regulating the encoding of (ir) relevant information in working memory (i.e., inhibitory deficit theory; Hasher, Lustig, & Zacks, 2007; Hasher & Zacks, 1988). Older adults' inhibitory deficits result in interference from irrelevant information and, consequently, impairment in WM performance (Gazzaley, Cooney, Rissman, & D'Esposito, 2005b; Schmitz, Cheng, & De Rosa, 2010; Zhu, Zacks, & Slade, 2010). Older adults, therefore, may be particularly impaired when processing emotional distractors that compete with other information. It is still unclear, however, whether age impacts the neural substrates underlying processing of positive and negative distractors.

While information processing in younger adults tends to favor negative stimuli (Baumeister, Bratslavsky, Finkenauer, & Vohs, 2001; Goldsmith & Dhar, 2013), older adults are more likely—than younger adults—to favor positive and less negative information in several cognitive domains, such as attention, memory, and decision-making (Brassen, Gamer, & Büchel, 2011; Mather & Carstensen, 2005; Ziaei, von Hippel, Henry, & Becker, 2015), known as the positivity effect (Mather & Carstensen, 2005; Reed, Chan, & Mikels, 2014). According to the cognitive control account, it is possible that effortful cognitive control to regulate negative emotions among older adults drives the positivity effect (Mather, 2012; Nashiro, Sakaki, & Mather, 2012). Our previous work supports this idea and further suggests that processing of negative items require greater cognitive control for older adults, reflected in greater activation of cognitive control areas (Ziaei, Salami, & Persson, 2017). Based on findings of a positivity effect in aging, we anticipate that older adults might have more difficulties in inhibiting positive information, relative to their younger counterparts. Several studies have supported this hypothesis by showing impaired performance from negative distractors in younger adults (Iordan & Dolcos, 2017). Other studies have also demonstrated less interference from negative distractors together with greater interference from positive distractors in older adults (Brassen et al., 2011; Ebner & Johnson, 2010; Goeleven, De Raedt, & Dierckx, 2010). For instance, one behavioral study using eye-tracker showed that positive distractors did not automatically capture attention of older adults when they were selectively directing their attention to negative items (Ziaei et al., 2015).

Given the inconsistencies in the literature and lack of understanding of this effect, in the current study, we examined the neural and behavioral substrates underlying the effect of emotional distraction in aging during a working memory task. Using a novel paradigm, we presented pairs of emotional-emotional or emotional-neutral pictures during encoding and participants were instructed to attend to either positive or negative items, while ignoring either emotional or neutral task-irrelevant information, during WM encoding (Ziaei, Peira, & Persson, 2014). Simultaneous presentation of the neutral or emotional (positive or negative) distractors with the emotionally valenced targets allows us to measure whether emotional distractors capture attention, as the extent to which distractors capture attention may vary as a function of age. The memory outcome for the targets and the effort expend during encoding of the targets may be differentially affected by the distractors’ valence. Second, based on the Socioemotional Selectivity Theory, “the critical contrast for the positivity effect is between positive and negative information” (Reed et al., 2014). Therefore, having emotionally valenced targets, relative to neutral targets, allows us to assess the memory positivity bias by measuring the discriminability of positive and negative distractors.

Moreover, brain regions involved in inhibition and/or interference resolution, such as the lateral PFC and striatum (Haeger, Lee, Fell, & Axmacher, 2015; Zhang, Geng, & Lee, 2017), are expected to be more engaged for emotional compared to neutral distractors. Consequently, emotional distraction might both disrupt ongoing processes relevant for WM encoding, as indicated by reduced activation in task-relevant fronto-parietal regions (Dolcos, Diaz-Granados, Wang, & McCarthy, 2008), while simultaneously increasing engagement in regions associated with emotional processes, such as the amygdala (Iordan et al., 2013).

Based on much converging evidence demonstrating age differences in neural engagement during inhibition (Gazzaley et al., 2005b), we also hypothesized that older and younger adults would engage different brain regions for inhibiting emotional compared with neutral distractors. Given our prior demonstrations of striatal and medial PFC activation for emotional compared with neutral distraction in younger adults (Ziaei et al., 2014), along with findings showing reduced striatal activation in older adults related to inhibition (Coxon et al., 2016), we hypothesized that older adults may show less activation in these regions. We also expected older adults to show more activation in frontal regions in response to emotional compared with neutral distractors, as suggested from theories of frontal-lobe compensation (Park & Reuter-Lorenz, 2009). Following the observations of age-related emotional biases, we also expected that younger and older adults might be differentially impaired in inhibiting negative and positive information, respectively. We hypothesized that in older adults, positive distractors would be associated with reduced performance and more disruption as indicated by lower activation in WM-related regions, such as ACC and lateral PFC compared with neutral and negative distractors. Similarly, in younger adults, positive distractors would be related to increased performance and less disruption as indicated by more activation in these regions compared with neutral and negative distractors. We also hypothesized that amygdala activation is more pronounced for positive distraction in older adults and negative distraction in younger adults.

## Materials and methods

### Participants

Sixteen younger adults and 15 older adults participated in this study. Two younger and two older participants were excluded from the analyses due to extensive head movement in the scanner and brain signal losses, leaving data from 14 younger adults (10 women; *M* = 22.64, *SD* = 1.69; range = 20-26 years) and 13 older adults (9 women; *M* = 68.42, *SD* = 3.8 years; age range = 64-74) reported in all analyses. Younger participants were recruited at Stockholm University, and older adults were recruited through flyers posted in libraries, churches, and hospitals. All participants were right-handed, Swedish speakers, with no history of neurological or psychiatric disorders. All participants were screened for claustrophobia, neurological and psychiatric medications, MRI contraindication, and all had normal or corrected normal vision. Additionally, older adults were screened for cognitive impairment using Mini Mental State Examination (MMSE; Folstein, Folstein, & McHugh, 1975), and all older adults scored at 26 or above (Table 1), indicating that all were cognitively intact. All participants agreed to take part in two separate sessions of testing: one for neuropsychological assessments and one for the fMRI scanning session. The investigation was approved by the Regional Ethical Review Board in Stockholm, and written, informed consent was obtained from all participants. Participants were paid 800 SEK (~US$ 96) for their participation.

**Table 1:** Demographics and cognitive performance for young and older adults

Demographics	Young adults	Older adults	*P*
N	14	13	
Age, years (range, SD)	22.6 (20-26, 1.7)	68.2 (64-74, 3.7)	
Gender (f/m)	10/4	9/4	n.s.
Education, years (range, SD)	2.7 (2-3, 0.5)	2.4 (1-3, 0.9)	n.s.
Cognitive scores			
Operation span	45.3 (20.1)	20.1 (13.4)	0.002
MMSE (range, SD)		27.6 (26-30, 1.2)	
Stroop task (RT)			
*Neutral (SD)*	830 (167.7)	1314.8 (184)	0.001
*Congruent (SD)*	856 (183.2)	1428 (261)	0.001
*Incongruent (SD)*	1070.5 (242.7)	1650.9 (308)	0.001

Values are means (range, SD) except for *gender*, which represents number of participants

MMSE, mini-mental state examination; *P, p* value for the comparison of young and older adults; RT, reaction time; n.s., nonsignificant

Education equals the number of years after high school

### Material

Stimuli consisted of colored pictures selected from the International Affective Pictures Systems (IAPS; Lang, Bradley, & Cuthbert, 2008). Pictures were rated as negative (valence: *M* = 2.83, *SD* = 1.7, arousal: *M* = 5.54, *SD* = 2.17), positive (valence: *M* = 6.79, *SD* = 1.73; arousal: *M* = 4.83, *SD* = 2.3), and neutral (valence: *M* = 4.87, *SD* = 1.26; arousal: *M* = 2.79, *SD* = 2.0). No significant differences were found in arousal levels of positive and negative stimuli (*p* > 0.05). Stimuli were presented in 600 × 800 pixels and were adjusted for the presentation in the scanner using E-prime software.

### Procedure

The study consisted of two sessions: 1) a behavioral testing session that took place in the Department of Psychology, Stockholm University, and 2) an fMRI session that took place at the MRI facility at the Karolinska hospital on a separate day. The fMRI session was conducted within a week from the behavioral session. During the behavioral testing session, participants completed the color-word Stroop test, the operation span working memory task (Unsworth, Heitz, Schrock, & Engle, 2005), and a test of visual attention (Bundesen, 1990). In addition, participants performed practice runs of the scanner task with a separate set of pictures until they were completely familiarized with the task. In the second testing session, and before scanning, participants performed one additional practice run. All participants completed a recognition memory test after the scanning session.

#### Emotional WM task

We used a modified version of a visual WM task developed by Gazzaley et al. (2005a, b) and a full description of the emotional WM task presented elsewhere (Ziaei et al., 2014). Participants first received an instruction either to attend to negative or positive pictures (5,000 ms). Then, three sequential screens each composed of a pair of pictures were presented (2,500 ms for each pair separated by a 500-ms fixation cross). Presentation of all three screens were followed by a fixation cross (maintenance; 4,000 ms) and finally a working memory probe (retrieval; 2,000 ms). Trials were separated using an inter-trial interval (ITI) that had a variable length (42% ITIs of 1.5 s, 28% ITIs of 3 s, 14% ITIs of 4.5 s, 12% ITIs of 6 s, and 4% ITIs of 7.5 s) that allows for an independent estimation of the BOLD response on a trial-to-trial basis. Simultaneous target–distractor presentation has the advantage of admitting us to measure whether emotional distractors capture attention and to what extent WM performance for distractors with different emotional valence vary as a function of age. To ensure that both the target and the distractor were noticed, a separate eye-tracking study was conducted (Ziaei et al., 2015). Results from this study showed that participants were indeed attending to the both the target and distractor images in order to make decision about which one to attend, and not only attended to the target by actively focusing their gaze towards (i.e., looking at) the relevant images, and looking away from the task-irrelevant images.

**Fig. 1:**
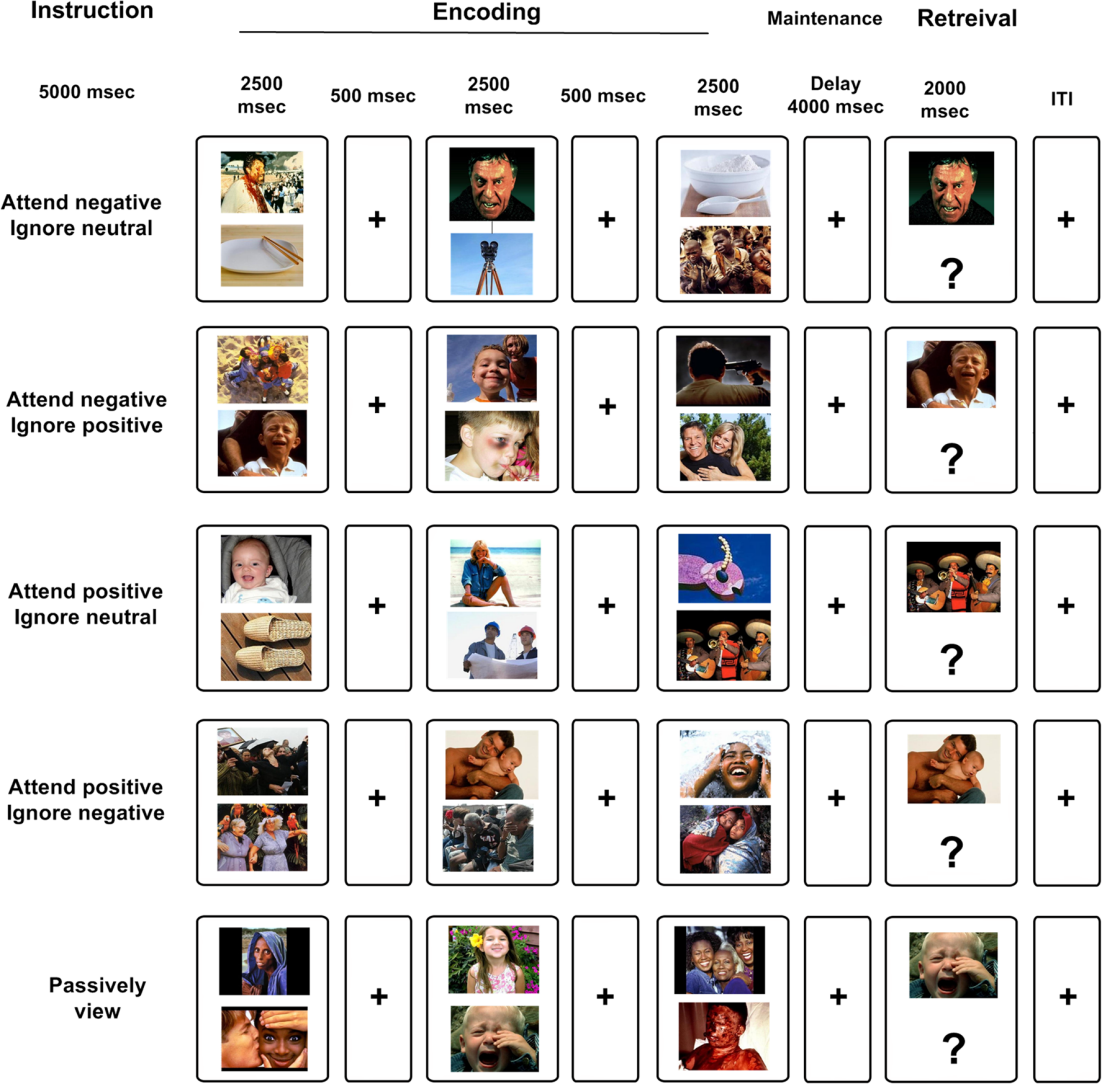
**Schematic overview of the fMRI working memory (WM) task with emotional distraction.** Functional magnetic resonance imaging (fMRI) data were recorded while participants performed a WM task with instructions to selectively attend to positive/negative targets and ignoring neutral or emotional (positive and negative) distractors. Working memory performance was measured using a yes/no forced-choice task where participants indicated with a button press whether the probe was part of the current target set. Stimuli consisted of pictures takes from the international affective picture system (IAPS) and were presented in color

In short, five different conditions were included in this fMRI experiment: (1) attend to negative pictures/ignore positive pictures, (2) attend to negative pictures/ignore neutral pictures, (3) attend to positive pictures/ignore negative pictures, (4) attend to positive pictures/ignore neutral pictures, (5) passively view the pictures (Fig. 1). During encoding, emotional/neutral (positive/neutral or negative/neutral) or emotional/emotional (positive/negative) pairs of pictures were presented and participants were asked to follow the instructions and direct their attentional focus to the relevant item/target and ignore the irrelevant item/distractor. During retrieval, an emotional picture/probe (with positive or negative valence based on the condition) was presented. Only positive and negative pictures were presented in the passive viewing condition, and participants were asked to attend to both pictures. During the retrieval phase, either a positive (50% of the trials) or negative picture was presented as a probe. Participants were instructed to respond with their right index finger if the probe matched one of the previously presented target pictures and press with their right middle finger if the probe did not match any of the previously seen pictures. In 50% of the trials, the probe was not matched with any of the targets. Note that we expected performance to be lower in the passive viewing condition, because all items are considered as targets (WM load = 6). Therefore, participants are required to attend to all presented items to successfully perform the task. In the instructed attention conditions, participants are required to attend selectively to emotional targets only, resulting in a lower WM load. Functional MRI data were collected in two separate runs, with each run containing 10 trials of each of the 5 conditions with a total time of approximately 30 min. All responses were recorded using a scanner compatible response box (Lumitouch, Inc.). The order of conditions was counterbalanced between participants.

#### Recognition memory task

After scanning, participants performed a self-paced recognition memory task that included pictures presented as targets during the emotional WM task intermixed with novel stimuli. A total of 130 pictures (100 previously shown pictures; 20 pictures from each condition) intermixed with 30 novel stimuli (10 positive, 10 negative, and 10 neutral pictures) were used for the recognition memory task. For each picture, participants were asked to indicate whether the picture had been presented previously during the WM task in the scanner, and also, for each picture, were asked to rate the confidence of their response using a 4-point scale (1 of 4 responses: sure old, unsure old, unsure new, sure new).

#### Positive and negative distraction index

To account for overall differences between age groups in the response to emotional distraction, a distraction index was used (similar to Grimshaw, Kranz, Carmel, Moody, & Devue, 2017; Wais & Gazzaley, 2014). For each younger and older participant, a distraction index was calculated for conditional correct WM scores (Supplemental Tables 1 and 2). The *positive distraction index* reflects the difference between ignoring positive information compared with ignoring neutral information, when participants attended to negative information (attend negative/ignore positive minus attend negative/ignore neutral). The *negative distraction index* reflects the difference between ignoring negative information compared with neutral information when participants attended to positive information (attend positive/ignore negative minus attend positive/ignore neutral). Positive and negative distraction index were calculated for three outcome measures: d’, RT, and BOLD signal (Supplementary Tables 1 and 2). Given that older adults often show a positivity bias, we hypothesized that they would perform better when asked to ignore negative distractors compared with ignoring neutral distractors while attending to positive targets. Given previous reports of a negativity bias in younger adults (Rozin & Royzman, 2001), we hypothesized that they would perform better when asked to ignore positive distractors compared with neutral distractors while they were instructed to attend to negative targets.

#### Image acquisition

Magnetic resonance imaging was performed using a 3-Tesla General Electric scanner MR750 equipped with a 32-channel head coil. Acquisition of functional data was achieved using a gradient echo-planar imaging sequence (37 transaxial slices, odd–even interleaved, 2 mm in plane resolution, thickness: 3.4 mm, repetition time [TR]: 2,000 ms, echo time [TE]: 30 ms, flip angle: 80°, field of view: 25 × 25 cm). To allow for progressive saturation of the fMRI-signal, 10 dummy scans were collected and discarded prior to experimental image acquisition. High-resolution T1-weighted structural images also were collected with a 3D fast spoiled gradient echo sequence (180 slices, with a 1-mm thickness, TR = 8.2 ms, TE = 3.2 ms, flip angle: 12°, field of view: 25 × 25 cm). The scanner task was presented to the participants on a computer screen, seen through a mirror mounted on the head coil, while the participant was lying in the scanner. Headphones and earplugs were used to dampen scanner noise, and cushions inside the head-coil helped to minimize head movements.

#### fMRI data preprocessing

All the fMRI data were analyzed within statistical parametric mapping software (SPM8, Welcome Department of Imaging Neuroscience, University College London, UK) implemented in Matlab 2010b (Mathworks Inc., MA). Following slice timing correction, motion correction was performed using the INRIAlign toolbox (Freire, Roche, & Mangin, 2002). Following coregisteration step, the “New Segment” procedure was used to segment each T1 image into gray matter (GM) and white matter (WM). The “DARTEL” toolbox was used to create a custom group template from the segmented GM and WM images (Ashburner, 2007). In addition, deformation from the group-specific template to each of the subject-specific GM/WM images was computed (i.e., flow field). Finally, the coregistered fMRI images and segmented GM/WM images were nonlinearly normalized, subject by subject, to the sample-specific template (using a subject-specific flow field), affine aligned into the Montreal Neurological Institute (MNI) template. Images were then resampled to 2 mm^3^ voxels and finally smoothed using an 8-mm FWHM Gaussian kernel.

At the end, voxel-level linear model was used to estimate and remove the global signal effects (Macey, Macey, Kumar, & Harper, 2004). The artifact repair toolbox (http://cibsr.stanford.edu/tools/human-brain-project/artrepair-software.html) was used to correct for the movement artefacts. None of the participants required more than 3% repair from all volumes.

#### fMRI data analyses

##### Whole-brain univariate analysis

Blocked (encoding phase), and event-related (retrieval phase) effects were modeled in the framework of the general linear model (GLM) as implemented in SPM8. However, given our specific interest in identifying the neural circuitry underlying processing of emotional distractors during encoding, we only used encoding phase data for the second level analysis. All regressors of interest were convolved with the hemodynamic response function. To account for in-scanner movement, three translational (x, y, z) and three rotational (pitch, roll, yaw) regressors obtained from the realignment step were included as covariates of no interest in the individual fixed effect analysis.

Single-subject statistical contrasts were set up using the general linear model, and group data were analyzed in a random-effects model. The main effect of instructed attention compared with passive viewing was analyzed in a 5 (condition) by 2 (age group) full factorial ANOVA in SPM. Regions derived from the main effect of instructed attention > passive viewing were used for subsequent ROI analyses (see below). For this contrast a familywise-error corrected (FWE) threshold of *p* < 0.05 was applied. For between-group analyses, contrast images (emotional distraction > neutral distraction) for each subject, generated using *t*-test, were taken into a second level between group analysis. For analyses of group differences, effects surviving an uncorrected threshold level of *p* < 0.001, with a cluster extent of >10 voxels were considered significant. Although methods for multiple comparison correction offers a conservative approach in controlling for type I errors, they are susceptible to type II errors (Lieberman & Cunningham, 2009). Given that we reported our findings in young adults group for this task (Ziaei et al., 2014), we expected to find increased activation in fronto-striatal regions in the contrast between instructed attention and passive viewing and that activation in these regions might differ between groups. Thus, the reason for using an uncorrected threshold for these contrasts were based on our a-priori hypotheses given the strong evidence implicating these regions in emotional distraction. Therefore, we believe that, given our a-priori hypotheses, the statistical and extent thresholds used in the current study provides a balance between type I and type II errors.

We also performed a follow-up analysis for the regions for which we had a-priori hypotheses where we first applied an uncorrected threshold of *p* < 0.001 and subsequently a family-wise (FWE) small-volume correction of *p* < 0.05. Both LIFG and ACC were significant at a small-volume correction of FWE <0.05. Small volume correction (SVC) was performed as implemented in SPM and based on the number of activated voxels within each particular cluster. All results are reported in MNI space.

#### Region-of-interest (ROI) analysis

We identified three regions for ROI analyses: the anterior cingulate cortex (ACC; -8 8 50), left inferior frontal gyrus (IFG; -40 20 26), and the left and right amygdala. Selection of each ROI was based on the importance of these regions in emotional processing, WM, and executive control based on prior literature (Brassen et al., 2011; Dolcos et al., 2008; Dolcos & McCarthy, 2006; Hart, Green, Casp, & Belger, 2010). For the cortical regions (ACC and IFG), we selected and defined regions that showed increased engagement during all instructed attention conditions compared to passive viewing (instructed attention > passive viewing) across all participants. Thus, we restricted the ROI analyses to significantly activated regions in this contrast. For this contrast a familywise-error corrected (FWE) threshold of *P* < 0.05 was applied. The left and right amygdala were anatomically defined by the AAL (anatomical automatic labelling) atlas using the WFU_Pickatlas toolbox (http://www.nitrc.org/projects/wfu_pickatlas/). The Marsbar toolbox (http://marsbar.sourceforge.net/) was used to create ROIs and to extract each ROI’s mean BOLD parameter estimate value for each condition for each participant. The parameter estimates were then used for plotting the results in SPSS, as well as for performing brain–behavior correlations. ROIs were functionally defined on the voxels that showed peak activations during instructed attention to emotion compared with passive viewing. Only regions that were identified a-priori were included in the ROI analyses, and further explored. Brain activation estimates were extracted by averaging BOLD signal (% signal change) from within a 10-mm sphere around the peak voxel for ACC and IFG. Effect sizes for the different conditions were then extracted and averaged across participants. All ROIs are depicted in Supplemental Table 1.

#### Statistical analysis

For the behavioural analyses, the average of the two conditions with emotional distraction (emotional distraction), and the average of the two condition with neutral distraction (neutral distraction) was used. For the ROI analyses, % signal change was extracted from each ROI and subjected to a mixed-model analysis of variance (ANOVAs) with age group as a between-subjects factor and target valence and distractor types as within-subjects’ factors using SPSS. Working memory accuracy was calculated as a discriminability index using d-prime scores, which represent the proportion of hit rates corrected for false positive rates (Snodgrass & Corwin, 1988). Median RTs were used in order to avoid excessive influence from deviant reaction times. For brain-behavior analyses, reaction times and accuracy were correlated with BOLD signal averaged from all voxels for emotional distraction conditions (conditions with either positive or negative distractors). Given the small sample size, we used Spearman’s rho, a nonparametric test of correlation.

## Results

### Behavioral results

#### Accuracy

**Fig. 2:**
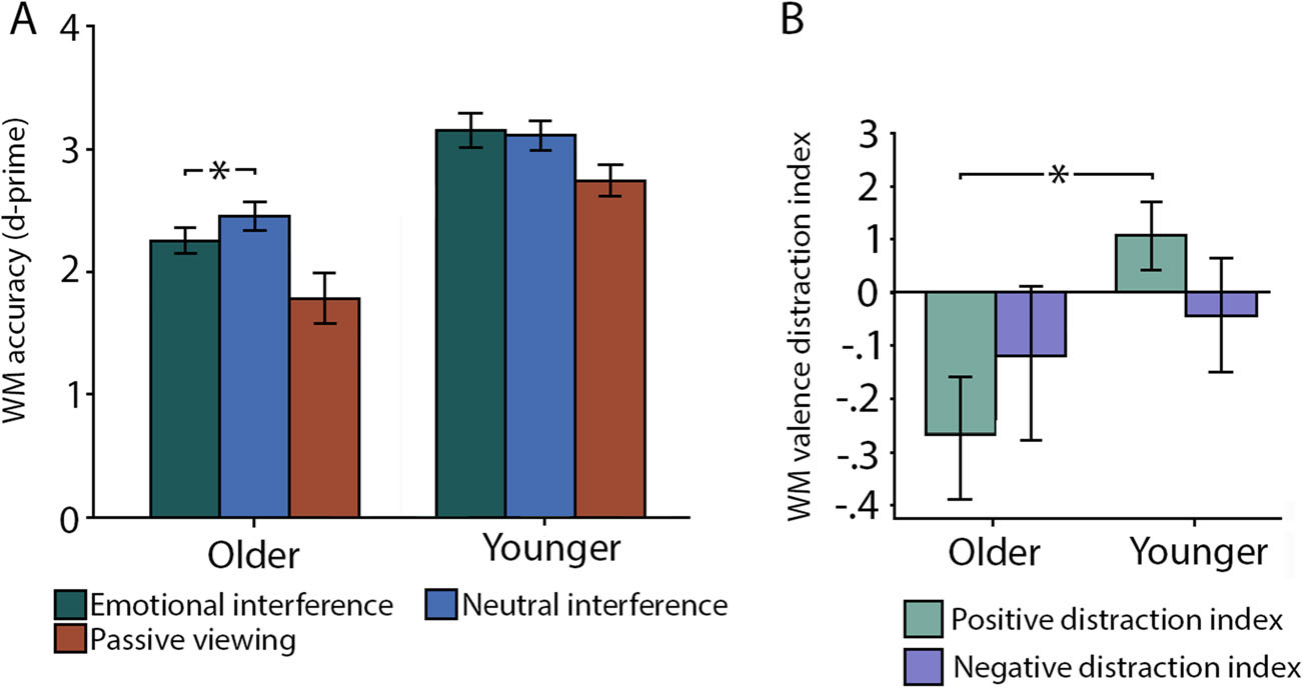
**Modulation of behavioral performance as a function of age and distractor valence.** (**A**) Working memory (WM) accuracy (d-prime) for emotional and neutral distraction, along with the control condition (passive viewing) for young and older adults respectively. Error bars indicate standard error of the mean (SEM). (**B**) Using a positive bias index, older adults showed differentially increased distractibility from positive distractors compared with neutral or negative distractors. Younger adults’ performance, conversely, was enhanced for positive distractors compared to neutral distractors

First, a 2 (condition: emotional vs. neutral distraction) × 2 (age group: younger vs. older adults) repeated measures ANOVA showed a main effect of age group (*F*(1,24) = 21.89, *p* < 0.001, η_p_^2^ = 0.49), where younger participants had higher performance compared with older adults. While the main effect of condition was not significant (*F*(1,24) = 1.88, *p* = 0.18, η_p_^2^ = 0.08), the condition by age interaction was marginally significant (*F*(1,24) = 4.31, *p* = 0.049, η_p_^2^ = 0.16), suggesting that older adults’ performance was lower when emotional distractors were presented compared with neutral distractors (*F*(12) = 5.46, *p* = 0.039, η_p_^2^ = 0.33 ). No such difference was found for younger adults (*F*(13) = 0.27, *p* = 0.61, η_p_^2^ = 0.02; Fig. 2A). Independent analyses showed that in older adults, performance was lower for emotional than neutral distractors (*F*(12) = 5.46, *p* = 0.039; η_p_^2^ = 0.33), whereas this difference was not significant for younger adults (*F*(13) = .27, *p* = 0.61, η_p_^2^ = 0.02).

Second, using the positive and negative distraction indices, we conducted a 2 (condition: positive and negative distraction index) × 2 (age group: younger vs. older adults) repeated-measures ANOVA showing nonsignificant main effects of condition and group (all *p*’s > 0.1). However, there was a significant condition by group interaction (*F* (1,24) = 5.67, *p* = 0.025, η_p_^2^ = 0.19), indicating that younger and older adults were differentially affected by the valence of the distractors. Follow-up analyses demonstrated a significant group difference between the positivity distractor index (*F* (1,24) = 8.37, *p* = 0.008, η_p_^2^ = 0.27), indicating that the memory performance for older adults was lower than younger adults’ when negative targets were presented with positive distractors. No between-group difference was found for the negative distractor index (*F* (1,24) = 0.30, *p* = 0.59, η_p_^2^ = 0.02), indicating that younger and older adults’ working memory performance did not differ for negative targets when they were asked to ignore negative distractors.

#### Reaction time

First, a 2 (condition: emotional vs. neutral distraction) × 2 (age group: younger vs. older adults) repeated measures ANOVA showed a main effect of age group (*F*(1,24) = 39.81, *p* < 0.001, η_p_^2^ = 0.61), with older adults responding slower compared with younger adults. However, the main effect of condition (*F*(1,24) = 0.25, *p* = 0.62, η_p_^2^ = 0.01), and the condition by group interaction were not significant (*F*(1,24) = 0.56, *p* = 0.46, η_p_^2^ = 0.02). Second, similar analyses were conducted on emotional valance distraction indices to investigate effects of differences in emotional distraction valence. No group differences were found for the negative distraction index, or the positive distraction index (all *p*s > 0.1) in reaction times.

#### Recognition memory performance

We further investigated whether instructed attention during encoding influenced off-line recognition memory performance. First, a 5 (all experimental conditions) by 2 (age groups; younger vs. older adults) repeated measure ANOVA analysis revealed a significant main effect of condition (*F*(4,88) = 4.52, *p* < 0.05, η_p_^2^ = 0.17), showing that recognition memory accuracy was higher for instructed attention conditions than passive viewing (all *ps* > 0.05). Neither the main effect of age nor the age by condition interaction was significant (all *p*s > 0.1).

### fMRI results

#### Whole-brain analysis: Main effect of emotional, compared with neutral distraction

First, the contrast of emotional compared to neutral distraction during WM encoding *across participants* showed significant activation in five regions; the right temporo-parietal cortex, the right precentral gyrus, the right inferior frontal gyrus, right posterior frontal gyrus, and the right putamen (Table 2). Given the strong hypothesis regarding fronto-striatal involvement during emotional distraction (Ziaei et al., 2014), a small volume correction (SVC) underscored the effect of emotional compared with neutral distraction in the right IFG and putamen (*p*_FWE_ < 0.05).

**Table 2:** MNI coordinates for areas that showed significant differences in the contrast of emotional distraction > neutral distraction across all participants

Anatomical localization	BA	x	y	z	mm^3^	*t*
Emotional distraction > neutral distraction						
R Middle temporal gyrus	39	52	−60	8	4424	4.03
R Superior frontal gyrus	6	22	−12	64	1064	3.70
R Middle frontal gyrus	9	46	24	26	864	3.35
R Inferior frontal gyrus	44	38	6	30	576	3.28
R Putamen		28	6	-8	112	3.11
Neutral distraction > emotional distraction						
R inferior frontal gyrus	46	26	32	14	3064	4.42
*R Anterior cingulate cortex*	*24*	*8*	*20*	*22*		*3.26*
Cerebellum		2	−54	-6	6344	4.14
L Medial frontal gyrus	32	−16	34	38	408	3.65
L Hippocampus		−18	−40	4	1000	3.49
L Anterior cingulate cortex	32	−18	34	10	304	3.42
R Caudate nucleus		18	−28	18	1696	3.39
R Parahippocampal gyrus		30	−28	-12	184	3.29
R Medial frontal gyrus	10	4	44	4	456	3.24

L, left; R, right; BA, Brodmann’s area; x, y, z, stereotactic coordinates

The opposite contrast, neutral compared to emotional distraction, revealed activation in right medial PFC, bilateral ventral ACC, right cerebellum, left medial dorsal frontal gyrus, left hippocampus, and right parahippocampal gyrus (Table 2).

#### Whole-brain analysis: Age differences in emotional compared with neutral distraction

**Fig. 3:**
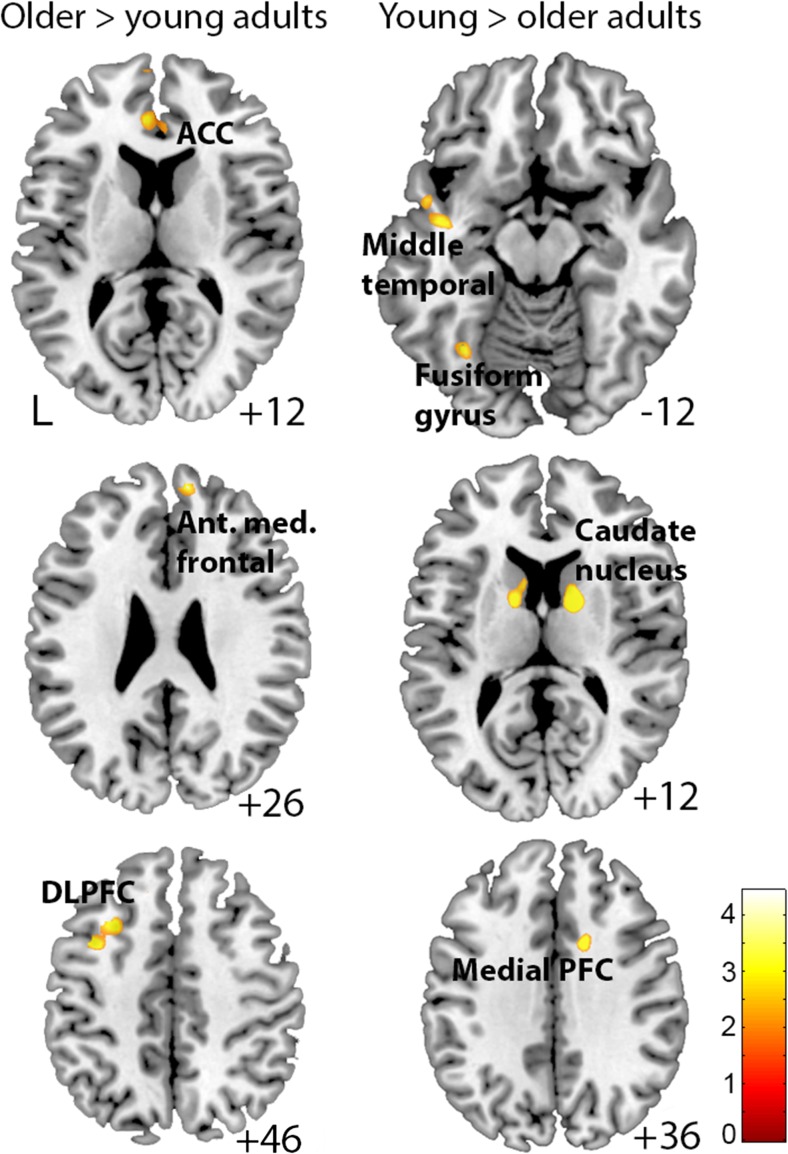
**Brain responses for emotional vs. neutral distraction as a function of age.** Blood oxygen level dependent response for emotional, compared with neutral distraction showing that older adults activated anterior medial prefrontal cortex, anterior cingulate cortex (ACC), the dorsolateral prefrontal cortex (DLPFC), and the left middle frontal/precentral gyrus more than younger adults. Younger, compared with older adults, activated the caudate nucleus, medial PFC, cerebellum, temporo-parietal cortex, fusiform gyrus, visual cortex, temporal gyrus, and bilateral hippocampus for emotional, compared to neutral distraction. The color bar indicates t-values

Second, age differences in emotional compared with neutral distraction were investigated. The results showed that older, compared with younger adults, activated primarily frontal regions, including left middle frontal/precentral gyrus, left dorsolateral PFC, ACC, and anterior medial PFC (Table 3; Fig. 3). Younger adults, on the other hand, showed increased activation in the caudate nucleus, medial PFC, cerebellum, temporo-parietal cortex, fusiform gyrus, visual cortex, temporal gyrus, and bilateral hippocampus, compared to older adults (Table 3; Fig. 3). Small volume corrections (SVC) underscored the age effects in left dorsolateral PFC, ACC (older > young), and caudate nucleus (young > older; *p*_FWE_ < 0.05).

**Table 3:** MNI coordinates for areas that showed significant age differences in the contrast of emotional distraction > neutral distraction

Anatomical localization	BA	x	y	z	mm^3 cluster size^	*t*
Older adults > younger adults						
L Middle frontal gyrus	6	−38	8	48	1048	4.51
*L Dorsal prefrontal cortex*	*9*	−*30*	*20*	*42*		*4.07*
L Anterior cingulate cortex	32	−4	40	12	1112	3.47
Younger adults > older adults						
R Posterior caudate nucleus		14	−4	16	1336	6.13
R Medial prefrontal cortex	24/32	14	10	36	632	4.74
R Posterior temporal cortex	37	66	−50	4	80	4.01
L Cerebellum		−16	−48	-10	264	3.86
L Middle temporal gyrus	21	−54	0	-8	776	3.78
*L Middle temporal gyrus*	*21*	−*42*	−*6*	*-14*		3.66
L Posterior caudate nucleus		−14	−4	-12	568	3.65
*L Caudate nucleus*		−*10*	*6*	*10*		*3.31*
L Fusiform gyrus		−34	−62	-14	256	3.49
L Lingual gyrus	17/18	−16	−76	-6	160	3.41
L Cingulate cortex	8	−20	−12	36	80	3.39
L Middle temporal gyrus	21	−52	−56	0	160	3.36
L Hippocampus		−34	−18	-12	160	3.29

L, left; R, right; BA, Brodmann’s area; x, y, z, stereotactic coordinates

#### ROI-analysis: Modulation of activation by emotional distractor valence and age

**Fig. 4:**
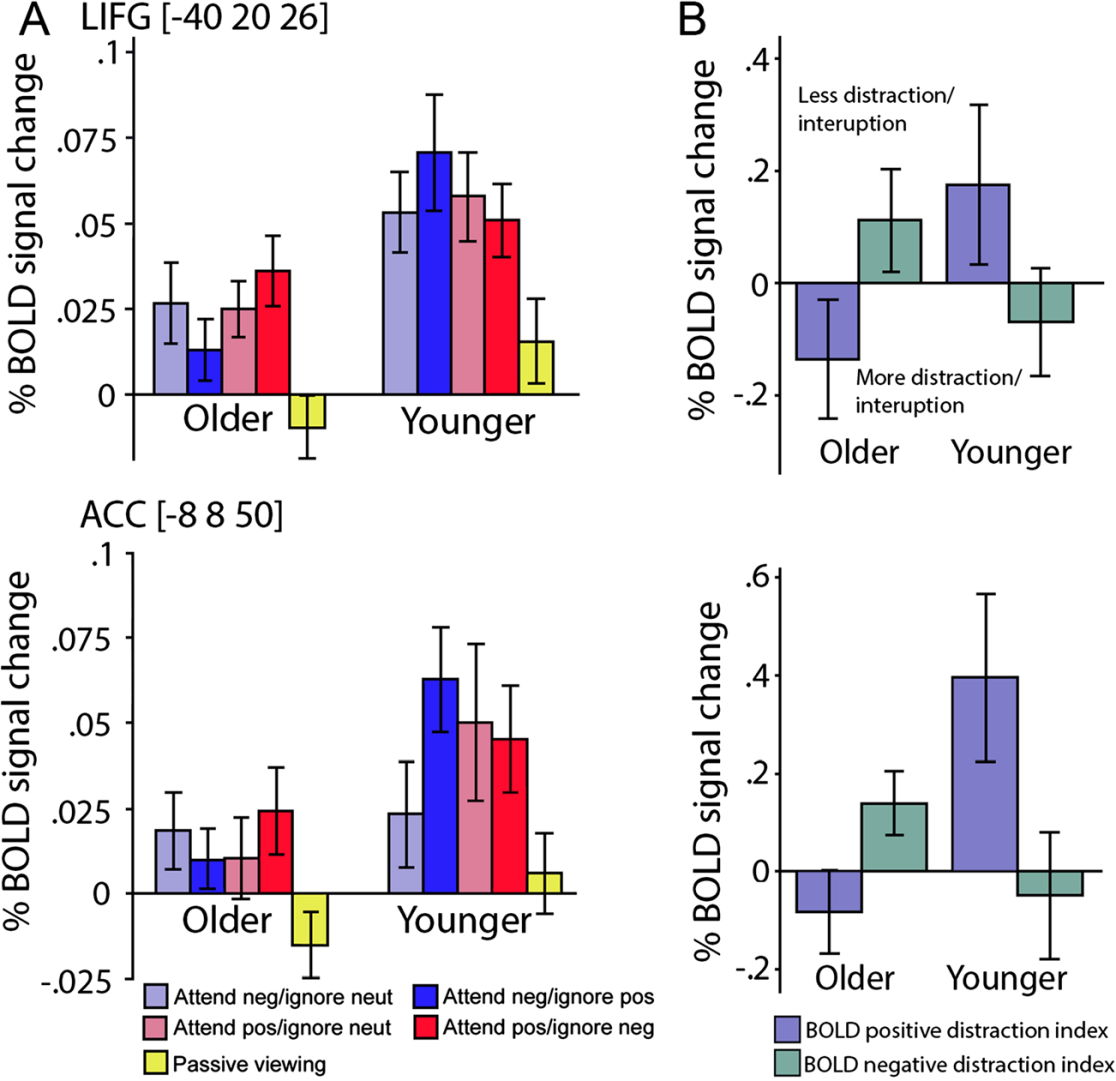
**Percent signal change in the left inferior frontal gyrus (IFG) and anterior cingulate cortex (ACC) as a function of condition and age.** (**A**) Blood oxygen level dependent response for each of the condition in the left (top) and ACC (bottom). (**B**) Using an emotional bias index, we showed that older adults were disproportionally affected, as indexed by reduced brain activation, for positive distractors compared to negative distractors. Activation in younger adults was enhanced for positive distractors compared to negative distractors. Error bars indicate standard error of the mean (SEM)

Third, we used an ROI approach that included the LIFG, ACC and amygdala to examine the interaction between emotional distractor’s valence and age. These results are shown in Fig. 4. Similar to the behavioral analyses, we investigated whether the valence of the distractors differentially modulated BOLD signal activation in younger and older adults. Activation in the left IFG showed a significant main effect of age (Fig. 4A; *F*(1, 24) = 5.54, *p* = 0.027, η_p_^2^ = 0.18), along with a significant age by condition interaction (Fig. 4A; *F*(1,24) = 2.85, *p* = 0.04, η_p_^2^ = 0.10). As indicated by the positive distractor index, for older adults, activation in this region was reduced when a positive distractor was presented compared with when a neutral distractor was presented (Fig. 4B; *F*(12) = 4.3, *p* = 0.048. η_p_^2^ = 0.15). Although non-significant, a tendency to the opposite pattern seemed to appear for younger adults (i.e., reduced activation for negative distractors; *F*(13) = 3.64, *p* = 0.08, η_p_^2^ = 0.25).

While we did not observe a reliable main effect of age in ACC activation (*F*(1,24) = 2.55, *p* = 0.123, η_p_^2^ = 0.09), the age by condition interaction was significant (Fig. 4A; *F*(1,24) = 3.01, *p* = 0.035, η_p_^2^ = 0.11). Similar to the results in the IFG, for older adults, activation in this region was reduced when a positive distractor was presented compared with when a neutral distractor was presented (Fig. 4B; *F*(12) = 5.14, *p* = 0.041, η_p_^2^ = 0.28), and an opposite pattern was found for younger adults (*F*(13) = 6.1, *p* = 0.021, η_p_^2^ = 0.20). For activation in the left and right amygdala, no main effects of age or condition were significant, neither were the age by condition interactions (all *p* > 0.1).

In sum, our results indicate that emotional distractor’s valence affect neural processing in the ACC and IFG. For positive distractors, these regions showed reduced activity among older adults and enhanced activity of among younger adults, relative to neutral distractors.

### Brain–behavior correlations

To examine further the relation between brain activity and individual performance on WM accuracy and RT, a brain-behavior correlation was conducted on the BOLD signal in the LIFG, ACC and amygdala with WM accuracy and RT. The results show that, across participants, activation in the ACC for emotional distraction was negatively correlated with d’ for emotional distraction conditions (*r* = −0.479, *P*_(uncorr.)_ = 0.013, *P*_(FDR)_ = 0.048), and activation in the LIFG for emotional distraction was positively correlated with d’ for emotional distraction conditions (*r* = 0.412, *P*_(uncorr.)_ = 0.036, *P*_(FDR)_ = 0.048). Moreover, activation in right amygdala during emotional distraction was positively correlated with d’ for emotional distraction conditions (*r* = 0.423, *P*_(uncorr.)_ = 0.031, *P*_(FDR)_ = 0.048). Differential brain–behavior correlations for these regions suggest that they may be involved in partly different cognitive operations during processing of emotional distractors. No other correlations between d’ and brain activations were significant (all *p* > 0.05). When RT was used as the behavioral measure, we observed significant positive correlations between activation during emotional distraction and RT for emotional distraction in the ACC (*r* = 0.584, *P*_(uncorr.)_ = 0.0013, *P*_(FDR)_ = 0.0026). In the LIFG, negative correlations were found for both emotional and neutral distraction conditions (emotional distraction: *r* = −0.616, *P*_(uncorr.)_ = 0.0006, *P*_(FDR)_ = 0.0024; neutral distraction: (*r* = −0.578, *P*_(uncorr.)_ = 0.0016, *P*_(FDR)_ = 0.0028). When the analyses were stratified by age group we found that for older adults, there was a negative relationship between LIFG activation during emotional distraction and RT for emotional distraction (*r* = −0.662, *P*_(uncorr.)_ =0.009, *P*_(FDR)_ = 0.036).

## Discussion

The present study was designed to investigate the neurobehavioral responses from emotional distraction in WM in younger and older adults. We demonstrate that WM accuracy was reduced in older adults when they were required to inhibit task-irrelevant emotional distractors compared with neutral distractors, whereas no such difference was found in younger adults. Younger and older adults also were differentially affected by the emotional valence of the distracting information. While older adults had lower performance for positive distractors, younger adults had higher performance, indicating that age specific emotional bias could influence the difficulty in suppressing task-irrelevant emotional information. Brain activity findings demonstrated that while younger adults activated the striatum for emotional compared to neutral distractors, older adults showed enhanced activation in lateral and medial prefrontal regions. In the left IFG and the ACC, brain activation findings corresponded to the behavioral age by valence interactions, suggesting that these particular regions are linked to processes that are differently involved in modulating attention to task-relevant emotional information, and away from task-irrelevant information depending on emotional bias. The fMRI findings are further strengthened by showing significant brain–behavior correlations. These findings are discussed in turn below.

### Modulation of behavioral performance by distractor valance and age

We did not find a main effect of emotional distractors compared to neutral distractors during WM encoding across participants. When age was considered, however, WM performance among older adults were found to be lower for emotional distractors compared with neutral distractors, suggesting a disproportional impairment that was elicited by emotional task-irrelevant information. Distractibility is considered a substantial concern in aging, and brain imaging observations have demonstrated insufficient distractor suppression in older adults as reflected by impaired cognitive performance and reduced neural responsiveness for task-irrelevant information (Chadick, Zanto, & Gazzaley, 2014; Clapp & Gazzaley, 2012; Gazzaley, Cooney, McEvoy, Knight, & D'Esposito, 2005a; Vaden, Hutcheson, McCollum, Kentros, & Visscher, 2012). Our results agree with these prior findings and extend these results by showing that emotional distraction may be more detrimental compared to neutral distraction and that this effect is mainly restricted to older adults for whom attentional resources may be reduced. Thus, in older adults, relative to the younger group, inhibition of emotional distractors seems to require more processing capacity that diminishes available resources for task-relevant operations, leading to impaired performance.

It is important to note that previous work have used different paradigms to examine attention to targets in the presence of emotional distractors. Older adults showed less interference from negative relative to positive items (Goelven et al., 2010), and their performance was affected by happy facial distractors (Brassen et al., 2011; Ebner & Johnson, 2010). Conversely, it has been demonstrated that older adult’s recognition memory performance is not affected by the presence of positive items (Ziaei et al., 2015). Altogether, it seems that using different methods and tasks might significantly affect how distractors are being processed by older adults. The use of different tasks, which might involve separate neural and cognitive processes, might at least partially explain the discrepancy between the current findings, and the lack of differential distraction effects for positive and negative items in older adults found in some previous studies (Ziaei et al., 2015). Further research is clearly needed to examine age-related changes in neural processing during encoding of emotionally-valenced relative to neutral distractors.

### Increased activation for emotional compared with neutral distractors across age groups

With regard to the fMRI results, BOLD signal increased in temporo-parietal, frontal, and striatal regions for emotional compared with neutral distractors across both age groups. These results suggest that emotional distractors, compared with neutral distractors, engage top-down processes, and adhere to the view that emotional stimuli tend to capture attention to a greater degree than nonemotional stimuli (Anderson, 2005; West, Anderson, & Pratt, 2009; Vuilleumier, 2005). The current results are in good agreement with previous findings showing increased striatal activation during inhibition of emotional nontargets compared with neutral nontargets in a Go/No-Go task (Hare, Tottenham, Davidson, Glover, & Casey, 2005) and the association of IFG and striatal activation for emotional interference during goal-directed selective attention (Papazacharias et al., 2015). The involvement of fronto-striatal regions in control of emotional distraction also is in line with findings from a recent meta-analysis on emotion-cognition interactions showing that IFG, putamen, and parietal regions are implicated in modulating the effect of emotion on cognitive control (Cromheeke & Mueller, 2014). This further strengthens the notion that these regions are key areas for control processes important for inhibiting task-irrelevant emotional information by counteracting the deleterious influence of emotional distractors on WM performance.

### Age differences in the neural response to emotional compared to neutral distraction

The neuroimaging findings showed that while younger adults recruited striatal and posterior cortical regions for emotion distraction conditions, older adults had significantly more activation in the prefrontal cortex. These results are in good agreement with results showing a posterior-anterior shift in aging (PASA), which is characterized by reduced activation in posterior brain sites, along with stronger activation in frontal regions in older compared with younger adults (Davis, Dennis, Daselaar, Fleck, & Cabeza, 2008; Maillet & Rajah, 2014; McCarthy, Benuskova, & Franz, 2014; Spreng, Wojtowicz, & Grady, 2010). A similar posterior–anterior shift in aging has been observed in emotional studies (Gunning-Dixon et al., 2003; St Jacques, Dolcos, & Cabeza, 2010; Tessitore et al., 2005), suggesting that this pattern is consistent across task domains. Prior findings, together with the current observations, provides evidence that the PASA pattern of activity can also be observed in emotional tasks. This pattern has typically been attributed to functional compensation for age-related impairments in cognitive processing and neural resources (Davis et al., 2008). This claim has been supported by findings that activity in frontal regions in older adults correlate positively with cognitive performance (Grady, McIntosh, & Craik, 2005; Heuninckx, Wenderoth, & Swinnen, 2008; Lighthall, Huettel, & Cabeza, 2014; Rajah & McIntosh, 2008). Our findings of a significant negative correlation between left IFG activation during emotional distraction and RT in older adults supports this view by indicating that older adults who perform better during emotional distraction also engage frontal regions to a larger extent.

### Modulation of brain activation by distractor valence and age

In addition to neurocognitive effects of emotional compared with neutral distraction, older adults’ behavioral performance was differentially modulated by the valence of distractors, i.e., their WM accuracy was lower for *positive* compared with *negative* distractors. The present results extend the previous literature on motivational disposition of older adults towards positive information (Carstensen, 2006), by showing that such a positivity bias can be unfavorable to performance if positive emotional information is presented as task-irrelevant distractors. This result is also in line with a previous finding using a spatial-cuing paradigm, in which they found that older adults had selectively increased distractibility from happy faces (Brassen et al., 2011) and that positive mood induction enhances encoding of task-irrelevant information (Biss, Weeks, & Hasher, 2012). Although we did not find any positivity effect in behavioral performance, it seems that the positivity effect can manifest itself differently based on whether positive information is presented as task-relevant targets or task-irrelevant distractors. Younger adults, on the other hand, showed a behavioral improvement when distractors had a positive, relative to negative valence, suggesting that they may exert less attentional resources to process positive information and more encoding resources could be allocated to successfully encode task-relevant negative information. The finding of a behavioral improvement for positive distraction compared with negative distraction in younger adults replicates a recent observation from a similar WM task in which emotional distraction was presented during the maintenance phase (Iordan & Dolcos, 2017). In line with the current results, Iordan and Dolcos (2017) were able to demonstrate that while negative distraction impaired performance, positive distraction resulted in enhanced performance. Together, our results show that WM performance can be modulated by the valence of the distractor, and that such modulation is age specific.

Interestingly, the age-related distraction effect was associated with a corresponding BOLD signal modulation in IFG and dorsal ACC, with lower activation in these regions for positive (compared with negative) distractors in older, relative to younger adults. Based on previous observations that emotional distractors, relative to neutral ones, can attenuate activity in task-relevant regions during the WM maintenance phase (Dolcos et al., 2008; Dolcos & McCarthy, 2006; Iordan & Dolcos, 2017; Iordan et al., 2013), we predicted that regions implicated in WM encoding may be disrupted by the presence of an emotional distractor. Our results are in line with this prediction. Both the IFG and ACC has been associated with WM and cognitive control in previous studies (Aron, Robbins, & Poldrack, 2014; Owen, McMillan, Laird, & Bullmore, 2005; Wager & Smith, 2003) and the current results indicate that processes subserved by these regions can be disrupted by emotional distractors. Consistent with this view, it has been demonstrated that emotional distractors transiently disrupt cognitive control (Kalanthroff, Cohen, & Henik, 2013; Pessoa, Padmala, Kenzer, & Bauer, 2012) and WM processes (Dolcos et al., 2008; Dolcos & McCarthy, 2006; Iordan & Dolcos, 2017; Shackman et al., 2006). It also was recently demonstrated that inducing a negative emotion (such as social threat) resulted in reduced WM performance and disrupted IFG and ACC activation (van Ast et al., 2016). Together, these results indicate that emotionally relevant information may attract resources available for the task and hence disrupt performance and task-relevant activation in the LIFG and ACC. Importantly, our results show that the amount of disruption is critically dependent on the valence of the distracting information and age; positive information is more disruptive for older adults, and negative information is more disruptive for younger adults. Moreover, significant brain–behavior correlations in the ACC and LIFG suggest that activation in these regions are related to different behavioral outcomes. While increased ACC activation was linked to worse performance, LIFG activation was associated with better performance. Differential brain–behavior correlations for these two regions indicate that they may be involved in partly different cognitive operations during processing of emotional distractors. One possibility, which is in line with much previous work (Barch, Braver, Sabb, & Noll, 2000; Botvinick, Braver, Barch, Carter, & Cohen, 2001; Carter & van Veen, 2007) is that the ACC monitors the occurrence of conflict between task-relevant and task-irrelevant information and subsequently conveys the information to other regions, such as IFG, to trigger control adjustments. Individuals that were able to maintain a high activation in these regions during WM encoding also performed better in the task, possibly by enhancing task-specific activation to maintain goal directed behavior during emotional distraction.

### Limitations

One limitation of the present study is the small sample size and that older adults most likely consisted of a group of high performing older adults compared with the general population. Future studies with larger sample sizes are needed to confirm our results. Also, it is most likely that the group of older adults constitute a group of comparatively high performing individuals and therefore may not be representative for the whole population of older adults. It also is plausible that older adults are a more select group compared to young adults, which may consist of individuals more typical for their age group. Furthermore, future studies should include neutral items as targets and compare the impact of emotional distractors when presented with neutral targets.

### Conclusions

The results of age-related differences in WM as a functional of emotional distractors adhere to the notion that a reduced ability to resist distraction may underlie impaired WM functioning in normal aging (Gazzaley et al., 2005b; Jost, Bryck, Vogel, & Mayr, 2011; McNab et al., 2015). Moreover, our results extend previous findings in two important ways. First, we demonstrated that reduced WM performance is not only affected by emotional distraction during maintenance of WM representations (Dolcos & McCarthy, 2006) but also when distracting information is presented during encoding and under selective attention instructions. Second, we showed that the extent to which emotional distractors impair WM performance and modulate task-relevant brain responses is contingent on the valance of the distractors, which varies as a function of age. These results are of particular relevance for the understanding of neurocognitive mechanisms associated with reduced ability to control emotional distraction, which are commonly observed in both aging and affective disorders.

## Electronic supplementary material

ESM 1 (DOCX 12 kb)

ESM 2 (DOCX 14 kb)
