# Supplementary material for: Age differences in the neural response to emotional distraction during working memory encoding
Source: Cogn Affect Behav Neurosci. 2018 Jun 11;18(5):869–83. doi: 10.3758/s13415-018-0610-8 (PMC6105189; doi:10.3758/s13415-018-0610-8)
Supplement: Supplementary file 1 — (DOCX 12 kb) [file 13415_2018_610_MOESM1_ESM.docx]

| Conditions | Performance accuracy (d’) [SD] | Distraction index | Diff. [SD] |
| --- | --- | --- | --- |
| *Younger adults* |  |  |  |
| Attend negative/ignore neutral | 3.12 [0.54] | Positive distraction index | 0.12 [0.33] |
| Attend negative/ignore positive | 3.24 [0.53] |  |  |
| Attend positive/ignore neutral | 3.11 [0.48] | Negative distraction index | -.05 [0.43] |
| Attend positive/ignore negative | 3.06 [0.61] |  |  |
| Passive viewing | 2.74 [0.44] |  |  |
| *Older adults* |  |  |  |
| Attend negative/ignore neutral | 2.51 [0.46] | Positive distraction index | -.27 [0.47] |
| Attend negative/ignore positive | 2.24 [0.37] |  |  |
| Attend positive/ignore neutral | 2.39 [0.56] | Negative distraction index | -.12 [0.60] |
| Attend positive/ignore negative | 2.27 [0.63] |  |  |
| Passive viewing | 1.78 [0.73] |  |  |

Positive distraction index = Attend negative/ignore positive – Attend negative/ignore neutral; Negative distraction index = Attend positive/ignore negative – Attend positive/ignore neutral; SD = Standard deviation.
