# Supplementary material for: Age differences in the neural response to emotional distraction during working memory encoding
Source: Cogn Affect Behav Neurosci. 2018 Jun 11;18(5):869–83. doi: 10.3758/s13415-018-0610-8 (PMC6105189; doi:10.3758/s13415-018-0610-8)
Supplement: Supplementary file 2 — (DOCX 14 kb) [file 13415_2018_610_MOESM2_ESM.docx]

| Conditions | Reaction times (in ms) [SD] | Distraction index | Diff. [SD] |
| --- | --- | --- | --- |
| *Younger adults* |  |  |  |
| Attend negative/ignore neutral | 957 [119] | Positive distraction index | 17 [71] |
| Attend negative/ignore positive | 975 [97] |  |  |
| Attend positive/ignore neutral | 936 [84] | Negative distraction index | 16 [109] |
| Attend positive/ignore negative | 951 [103] |  |  |
| Passive viewing | 1019 [107] |  |  |
| *Older adults* |  |  |  |
| Attend negative/ignore neutral | 1268 [214] | Positive distraction index | -16 [133] |
| Attend negative/ignore positive | 1252 [165] |  |  |
| Attend positive/ignore neutral | 1265 [193] | Negative distraction index | -6 [99] |
| Attend positive/ignore negative | 1259 [157] |  |  |
| Passive viewing | 1277 [208] |  |  |

Positive distraction index = Attend negative/ignore positive – Attend negative/ignore neutral; Negative distraction index = Attend positive/ignore negative – Attend positive/ignore neutral; SD = Standard deviation. ms = milliseconds.
